# Percutaneous Left Ventricular Unloading During High-Risk Coronary Intervention: Rationale and Design of the CHIP-BCIS3 Randomized Controlled Trial

**DOI:** 10.1161/CIRCINTERVENTIONS.123.013367

**Published:** 2024-02-27

**Authors:** Matthew Ryan, Saad M. Ezad, Ian Webb, Peter D. O’Kane, Matthew Dodd, Richard Evans, Lynn Laidlaw, Sohail Q. Khan, Roshan Weerackody, Alan Bagnall, Vasileios F. Panoulas, Haseeb Rahman, Julian W. Strange, Farzin Fath-Ordoubadi, Stephen P. Hoole, Rod H. Stables, Nick Curzen, Tim Clayton, Divaka Perera

**Affiliations:** School of Cardiovascular and Metabolic Medicine & Sciences at the British Heart Foundation Centre of Research Excellence, King’s College London, United Kingdom (M.R., S.M.E., H.R., D.P.).; King’s College Hospital National Health Service (NHS) Foundation Trust, London, United Kingdom (I.W.).; University Hospitals Dorset NHS Foundation Trust, Bournemouth, United Kingdom (P.D.O.).; Clinical Trials Unit, London School of Hygiene & Tropical Medicine, United Kingdom (M.D., R.E., T.C.).; Patient and Public Contributor, London School of Hygiene & Tropical Medicine, United Kingdom (L.L.).; University Hospitals Birmingham NHS Foundation Trust, United Kingdom (S.Q.K.).; Bart’s Health NHS Foundation Trust, London, United Kingdom (R.W.).; Newcastle Hospitals NHS Foundation Trust (A.B.).; Harefield Hospital, London, United Kingdom (V.F.P.).; University Hospitals Bristol NHS Foundation Trust, United Kingdom (J.W.S.).; Manchester University Hospitals NHS Foundation Trust, United Kingdom (F.F.-O.).; Royal Papworth Hospital NHS Foundation Trust, Cambridge, United Kingdom (S.P.H.).; University of Liverpool, United Kingdom (R.H.S.).; University of Southampton, United Kingdom (N.C.).; Guy’s and St Thomas’ NHS Foundation Trust, London, United Kingdom (D.P.).

**Keywords:** coronary artery disease, heart-assist devices, left ventricular systolic dysfunction, percutaneous coronary intervention, randomized controlled trial

## Abstract

**INTRODUCTION:**

Percutaneous coronary intervention for complex coronary disease is associated with a high risk of cardiogenic shock. This can cause harm and limit the quality of revascularization achieved, especially when left ventricular function is impaired at the outset. Elective percutaneous left ventricular unloading is increasingly used to mitigate adverse events in patients undergoing high-risk percutaneous coronary intervention, but this strategy has fiscal and clinical costs and is not supported by robust evidence.

**METHODS:**

CHIP-BCIS3 (Controlled Trial of High-Risk Coronary Intervention With Percutaneous Left Ventricular Unloading) is a prospective, multicenter, open-label randomized controlled trial that aims to determine whether a strategy of elective percutaneous left ventricular unloading is superior to standard care (no planned mechanical circulatory support) in patients undergoing nonemergent high-risk percutaneous coronary intervention. Patients are eligible for recruitment if they have severe left ventricular systolic dysfunction, extensive coronary artery disease, and are due to undergo complex percutaneous coronary intervention (to the left main stem with calcium modification or to a chronic total occlusion with a retrograde approach). Cardiogenic shock and acute ST-segment–elevation myocardial infarction are exclusions. The primary outcome is a hierarchical composite of all-cause death, stroke, spontaneous myocardial infarction, cardiovascular hospitalization, and periprocedural myocardial infarction, analyzed using the win ratio. Secondary outcomes include completeness of revascularization, major bleeding, vascular complications, health economic analyses, and health-related quality of life. A sample size of 250 patients will have in excess of 80% power to detect a hazard ratio of 0.62 at a minimum of 12 months, assuming 150 patients experience an event across all follow-up.

**CONCLUSIONS:**

To date, 169 patients have been recruited from 21 National Health Service hospitals in the United Kingdom, with recruitment expected to complete in 2024.

**REGISTRATION:**

URL: https://www.clinicaltrials.gov; Unique identifier: NCT05003817.

**WHAT IS KNOWN**

- Percutaneous left ventricular assist devices are increasingly used to support high-risk coronary intervention procedures.
- Observational studies examining the association between percutaneous left ventricular assist device use and outcomes have shown divergent results.
- There is no randomized controlled trial evidence comparing the use of left ventricular unloading with the current standards of care.

**WHAT THE STUDY ADDS**

- CHIP-BCIS3 (Controlled Trial of High-Risk Coronary Intervention With Percutaneous Left Ventricular Unloading) will provide high-quality evidence on the use of percutaneous left ventricular unloading in high-risk coronary intervention.

The development of cardiogenic shock in a previously stable patient is a devastating consequence of percutaneous coronary intervention (PCI) and often results in death or life-changing morbidity, prolonged hospitalization, and increased resource expenditure.^1^ Developments in technology and technique allow revascularization to be offered to an increasingly comorbid population with complex coronary disease. Although these patients stand to gain most from PCI, they are also at the highest risk. Shock may develop abruptly due to overt complications or insidiously as a consequence of cumulative ischemia. The risk is the highest in patients with impaired cardiac function at the outset, which reduces the capacity of the left ventricle (LV) to withstand further ischemic insults.^2–4^ Several anatomic and procedural factors are associated with increased risk; the primary anatomic determinant is the extent of coronary disease and associated volume of jeopardized myocardium, exemplified by patients with left main stem or multivessel coronary disease.^5–7^ Technical factors associated with early adverse events include the need for calcium modification, bifurcation stenting, and retrograde access to chronic total occlusions.^8–13^

The desire to avoid procedural complications often leads to patients at high risk either not undergoing intervention or receiving revascularization of lower extent and quality, both of which are associated with increased morbidity and mortality.^14^ The quest to prevent shock and facilitate more effective revascularization in this population is important but remains incompletely realized: mechanical circulatory support (MCS) is regarded as one of the most promising solutions.

## CONTEMPORARY MCS STRATEGIES

Circulatory support devices can be beneficial via 2 mechanisms: minimizing ischemia and maintaining cardiac output if ischemia ensues. The devices most often used are those that can be inserted percutaneously in the cardiac catheterization laboratory. While MCS can also be used to treat established shock, the focus of this trial is on nonemergent high-risk PCI.

The first MCS strategy to be subjected to randomized evaluation in high-risk PCI was the intra-aortic balloon pump (IABP). In BCIS-1 (Balloon Pump Assisted Coronary Intervention Study), patients with an LV ejection fraction ≤30% and a high British Cardiovascular Intervention Society Jeopardy Score were randomized to have PCI with elective IABP support or PCI with no planned IABP (Table).^15^ At hospital discharge, there was no difference in the rate of major adverse cardiac and cardiovascular events between groups.^16^ Patients assigned to have PCI without IABP support had more intraprocedural complications (necessitating bailout IABP use in 13% of cases), but the group assigned to elective IABP had more bleeding and access site complications. Consequently, contemporary guidelines recommend against routine IABP insertion in patients undergoing high-risk PCI.^17,18^ At long-term follow-up, a 33% reduction in all-cause mortality was observed in patients assigned to elective IABP support. While the mechanism is unclear, it has been hypothesized that this may relate to a reduction in periprocedural myocardial injury, the effects of which may only manifest in the longer term.^19^ The potential this raises for detection of late effects is important in planning future studies of MCS devices. IABP remains widely used as a bailout device in patients who develop shock during PCI.

**Table.**
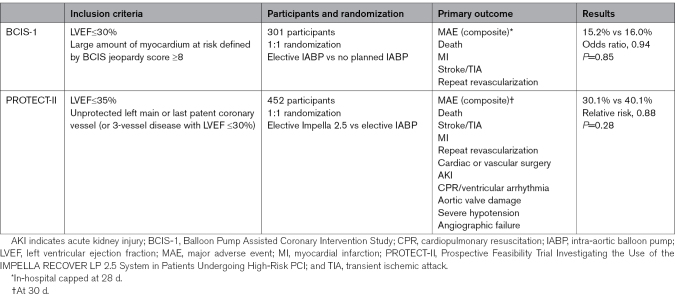
Randomized Trials of Mechanical Circulatory Support in High-Risk Percutaneous Coronary Intervention

Since the publication of the BCIS-1 trial, the use of IABP to support high-risk PCI has remained infrequent. In contrast, there has been a progressive increase in the use of the percutaneous LV assist device (pLVAD), which is now the most commonly used MCS device during high-risk PCI in the United States.^20,21^ A pLVAD is a catheter-mounted transvalvular microaxial flow pump that is placed retrogradely across the aortic valve and unloads the LV by drawing blood continuously into the aorta. The Impella (Abiomed, Danvers, CA) is the only pLVAD currently licensed for use in the United Kingdom and United States. Observational clinical and animal data have confirmed that these devices have potent hemodynamic effects, with reduction in LV work, and hence myocardial oxygen demand, as well as possible beneficial effects on coronary perfusion.^22–24^ However, to date, there has been no randomized evaluation of the safety and efficacy of elective pLVAD support during high-risk PCI, compared with unsupported PCI.

The only randomized evaluation of pLVAD-supported high-risk PCI was the PROTECT-II trial (Prospective Feasibility Trial Investigating the Use of the IMPELLA RECOVER LP 2.5 System in Patients Undergoing High-Risk PCI), in which the comparator arm was IABP-supported PCI (Table).^25^ This trial was terminated prematurely (as the Data and Safety Monitoring Committee _identified futility in showing efficacy for the primary outcome), and consequently only 442 of 600 intended patients were randomized. There was no significant difference between groups in the occurrence of the 10-component composite primary outcome or major adverse events at either 30 or 90 days by intention-to-treat analysis. In a secondary per-protocol analysis at 90 days, the group assigned to Impella experienced significantly fewer primary outcome events. This was driven mostly by a reduction in less clinically significant events including repeat revascularization and periprocedural hemodynamic instability, while the occurrence of death and myocardial infarction (MI) was numerically higher in the group treated with Impella.

There have been several observational comparisons of pLVAD-supported PCI with either IABP-supported PCI or unsupported PCI, some of which have used techniques such as propensity matching in a bid to overcome differences in patient characteristics (Table S1). The results have been discrepant. An initial study from the Premier Healthcare Database indicated increased odds of death, bleeding, and stroke in patients treated with the Impella device,^20^ while the NCDR study (National Cardiovascular Data Registry) reported lower rates of major adverse cardiac events but a higher rate of procedural complications.^21^ An updated study from the Premier Healthcare Database, which examined a narrower time window from 2016 to 2019 and looked exclusively at patients undergoing nonemergent PCI procedures, found that Impella-supported PCI was associated with lower odds of in-hospital mortality, cardiogenic shock, and subsequent MI than IABP-supported PCI with no difference in stroke, bleeding, or vascular complications.^26^ The bias and confounding inherent in these observational data limit their interpretation, although the divergent results emphasize the knowledge gap that exists. Higher quality evidence is therefore needed to support the wider adoption of these techniques into clinical practice.

### Complications of pLVAD Use

The most frequent complications of pLVAD use relate to large-bore vascular access. Vascular complications have particular consequences for patients undergoing high-risk PCI, as bleeding, anemia, transfusion, and the need for emergency vascular surgery are associated with a markedly elevated risk of stent thrombosis and worsening heart failure.^27^ A recent systematic review reported a 5.5% risk of major vascular complications and need for blood transfusion in patients undergoing pLVAD-supported high-risk PCI.^28^ While greater institutional and operator experience with large-bore access from transcatheter aortic valve intervention, preprocedural imaging of the peripheral vasculature, and novel strategies for device-based percutaneous vascular closure might reduce bleeding complications, it is unknown whether these developments translate into improved clinical outcomes. The risk of complications reinforces the need for objective evidence that the benefit of LV unloading outweighs the risks specific to this population.

### International Practice Guidelines on pLVAD-Supported PCI

**Figure 1.**
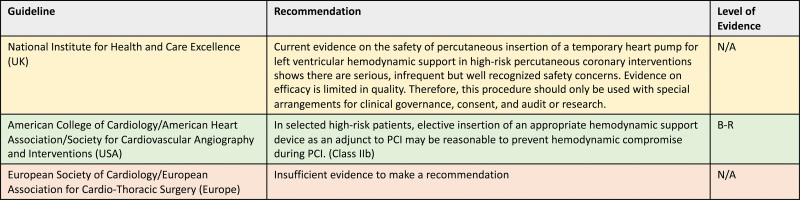
**Contemporary guidelines on the use of mechanical circulatory support in high-risk percutaneous coronary intervention (PCI).** The National Institute for Health and Care Excellence guideline committee does not provide a summary estimate of the level of evidence but acknowledged the lack of high-quality evidence. B-R indicates level of evidence B, one or more randomized trials; N/A, not available; and UK, United Kingdom.

Current clinical guidelines provide divergent recommendations (Figure 1). In 2018, the UK National Institute for Health and Care Excellence concluded that “the current evidence on the safety of percutaneous insertion of a temporary heart pump for LV hemodynamic support in high-risk PCI shows there are serious, infrequent but well recognized safety concerns” and “evidence of efficacy is limited in quality.”^29^ The report concluded that the procedure should only be used with special arrangements for governance, consent, and audit or research. The 2021 American College of Cardiology/American Heart Association/Society for Cardiovascular Angiography and Intervention guideline for coronary artery revascularization recommends elective MCS may be reasonable in selected patients but acknowledges the lack of high-quality evidence to support this position.^18^ The 2018 European Society of Cardiology guideline on myocardial revascularization recommends against routine use of the IABP but makes no recommendation on the use of percutaneous LV unloading for high-risk PCI, due to a lack of evidence.^17^

In response to the need for data identified by the National Institute for Health and Care Excellence and the international community, we developed CHIP-BCIS3 (Controlled Trial of High-Risk Coronary Intervention With Percutaneous Left Ventricular Unloading). The trial aims to provide a randomized comparison between percutaneous LV unloading and the current standard of care for high-risk PCI, as defined in BCIS-1. The target population will be carefully phenotyped patients who are at the highest risk for ischemic complications, with only a few exclusion criteria to ensure generalizability of the results. Further, we will report complete and transparent data, with a primary outcome that combines both efficacy and safety outcomes for an overall assessment of the benefits of this technology, including analysis of cost-effectiveness. Finally, given the evidence of a late benefit of MCS in both BCIS-1 and PROTECT-II, we aim to provide longer term follow-up within the primary analysis.

## METHODOLOGICAL CHALLENGES IN DESIGNING HIGH-RISK PCI TRIALS

### Patient Population and Randomization

Despite decades of research, no universally accepted definition exists for high-risk PCI. Several definitions and scoring systems have been proposed, which seek to integrate clinical, coronary, LV, and technical aspects to predict elevated procedural risk, with varying degrees of validation, but none have been universally adopted.^13,30–34^ In addition to identifying patients who are most likely to benefit from MCS, determining the ideal trial population requires consideration of feasibility and generalizability. The choice of participating centers is important: recruiting centers need to have high-volume complex PCI programs, experience of pLVAD device deployment, expertise in large-bore arterial access, and a track record of recruitment to randomized clinical trials, while the use of pLVAD devices must not be so engrained that a perceived lack of equipoise prevents either the recruitment of consecutive patients or reduces the confidence of operators in safely delivering interventions without the device, which could lead to a high crossover rate.

### Periprocedural MI

The definition and relevance of periprocedural MI in revascularization trials is contentious.^35^ As in routine clinical practice, there is a trade-off between sensitivity and specificity, largely relating to the binary biomarker thresholds used to define periprocedural MI. The Fourth Universal Definition of Myocardial Infarction is most widely accepted and endorsed by the American College of Cardiology, American Heart Association, European Society of Cardiology and World Health Organization, but is poorly specific for future adverse events and lacks validation in patients with elevated biomarker levels at baseline, a situation that is commonly encountered in high-risk PCI.^36^ Alternative definitions (such as those provided by the Society for Cardiovascular Angiography and Interventions and Academic Research Consortium) provide criteria for adjudicating MI in the context of elevated or rising levels but are not as universally accepted, and their higher thresholds for diagnosis are much less sensitive in predicting future adverse events.^37,38^ Clinical criteria used in all 3 definitions do not provide greater prognostic accuracy compared with solely biomarker-based definitions and are more subjective and prone to incomplete capture.^39,40^

Periprocedural MI is used as a surrogate of prognostically important clinical outcomes such as cardiovascular hospitalization and death but when combined with the latter in a composite primary outcome measure, it may obscure more significant events. When using traditional time-to-first-event analyses, subsequent events are discarded and periprocedural MI (which occurs early) may, therefore, have a greater impact on the composite than later hard outcomes. A final consideration is that it is most often adjudicated as a binary outcome, which fails to capture the powerful prognostic impact of the magnitude of periprocedural MI.^35,40^ This is particularly relevant in evaluating strategies to mitigate periprocedural ischemia and MI, as is the case in CHIP-BCIS3.

### Choosing and Analyzing Outcome Measures

Traditional composite primary outcomes combine adverse events of varying degrees of severity, often ranging from death to clinically minor events, all of which are given equal weight in the analysis. This issue is compounded by time-to-first-event analysis of revascularization trials, as discussed above. Combining efficacy and safety end points is also challenging and can complicate interpretation where there are both efficacy and safety differences within the same study. This type of analysis inadequately reflects the priorities of both patients and investigators and can hide important later signals of mortality benefit.

The win ratio is an increasingly recognized method of statistical analysis that has the potential to address many of the above limitations. The method was developed at the London School of Hygiene & Tropical Medicine and is increasingly used in cardiovascular randomized trials.^41–44^ Components of a composite outcome measure are treated according to a prespecified hierarchy, which weights outcomes in terms of clinical importance and of impact to patients, allowing the integration of efficacy and safety outcomes. The analysis of results uses an unmatched comparison, where each participant in one randomized group is compared with every participant in the other treatment group, with follow-up time for each comparison capped at the shortest duration of follow-up within that pair. As the method involves unmatched comparisons, every potential pair of patients within the trial is included, resulting in a large number of comparisons. Furthermore, the analysis is dynamic over the course of trial follow-up and is updated when new events occur. Hence more important outcomes that may occur later are given proper weight. The method also allows the integration of continuous variables into the primary outcome, such as the magnitude of periprocedural MI. The win ratio is particularly suited to clinical scenarios like high-risk PCI, where event rates for hard clinical outcomes are expected to be high, as opposed to trials with lower event rates where softer outcomes may dominate the analysis.

In summary, the method prioritizes events that are meaningful to patients and clinicians and is particularly suited to strategy trials involving devices, where the intervention is performed at the outset and there is a low risk of crossover.

## TRIAL DESIGN

**Figure 2.**
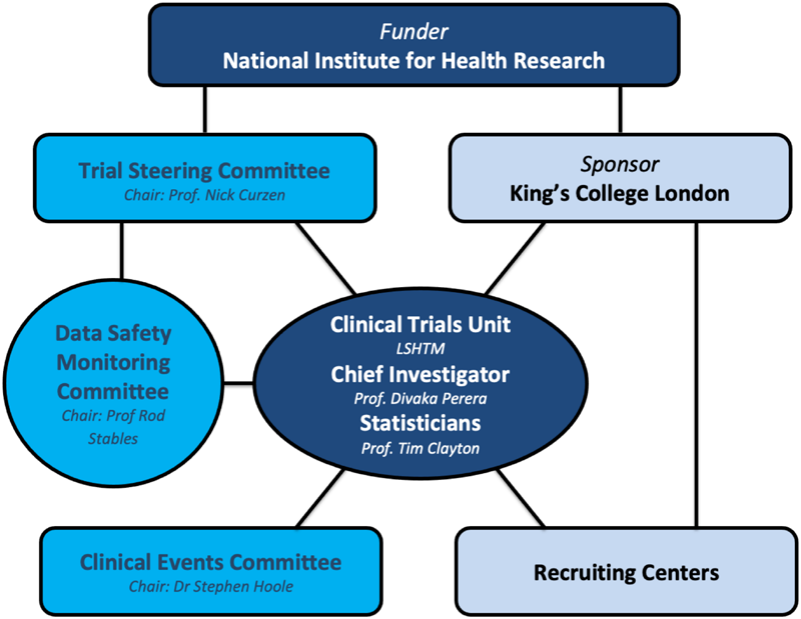
**Trial organization.** LSHTM indicates London School of Hygiene & Tropical Medicine.

CHIP-BCIS3 is a prospective, multicenter, open-label randomized controlled trial. The primary hypothesis is that a strategy of elective percutaneous left ventricular unloading during high-risk PCI procedures improves clinical outcomes and quality of life and is cost-effective. The trial is funded by the National Institute for Health Research Health Technology Assessment Program, sponsored by King’s College London and Guy’s and St Thomas’ National Health Service (NHS) Foundation Trust, coordinated by the London School of Hygiene & Tropical Medicine Clinical Trials Unit and endorsed by the British Cardiovascular Intervention Society. Independent trial steering committee and data monitoring committees oversee the progress of the trial (Figure 2; Table S2).

The trial is recruiting at NHS hospitals in the United Kingdom. All centers and operators have established high-volume complex PCI programs, extensive experience with large-bore arterial access (from pLVAD, transcatheter aortic valve intervention, or endovascular aneurysm repair), and track records of research delivery (Table S3). These measures were taken to ensure optimal performance of the pLVAD implantation and PCI procedure. This may limit the generalizability of the results to low volume centers, a setting in which complex high-risk procedures are more rarely performed.

### Inclusion/Exclusion Criteria

Inclusion and exclusion criteria were developed using a Delphi process whereby 6 expert complex PCI operators were invited by the Chief Investigator to provide individual feedback on proposed inclusion criteria in 3 rounds before a consensus was developed.^45^

**Figure 3.**
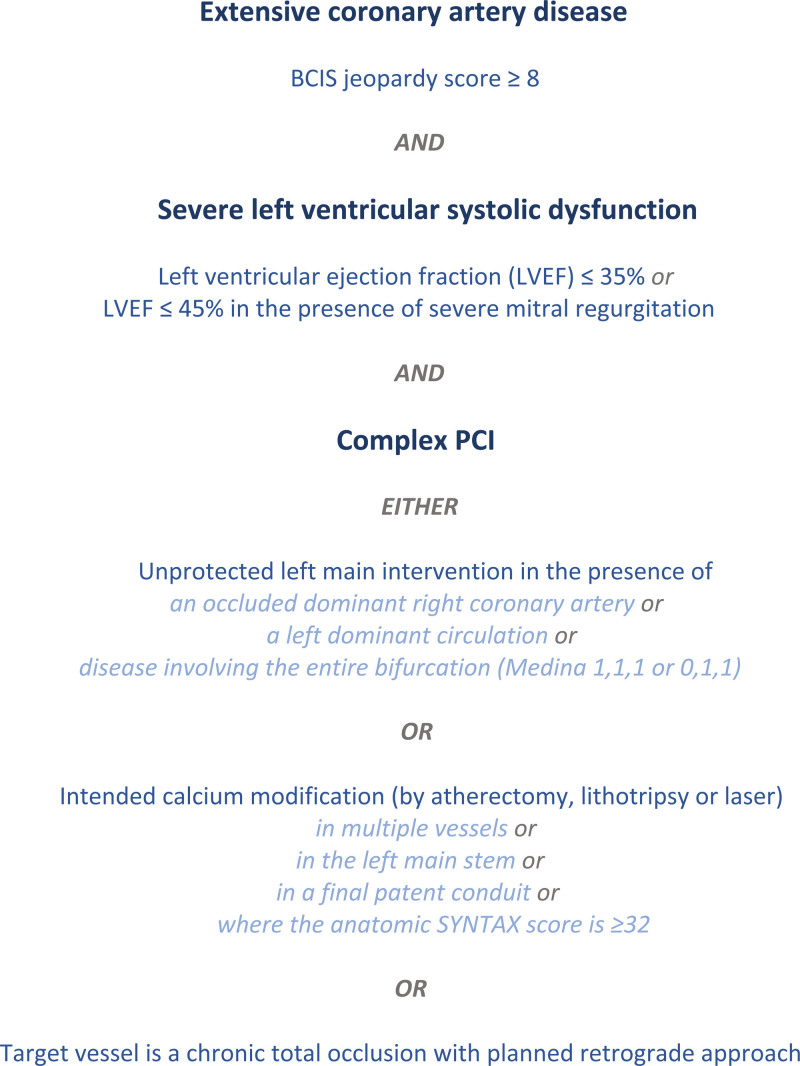
**Inclusion criteria for CHIP-BCIS3 (Controlled Trial of High-Risk Coronary Intervention With Percutaneous Left Ventricular Unloading).** BCIS indicates British Cardiovascular Intervention Society; LVEF, left ventricular ejection fraction; PCI, percutaneous coronary intervention; and SYNTAX, Synergy Between PCI With Taxus and Cardiac Surgery.

Patients are eligible for inclusion if they have extensive coronary artery disease, severe LV systolic dysfunction, and are scheduled to undergo complex PCI (Figure 3). Qualifying assessments of LV ejection fraction must have been performed within 1 year before randomization, and coronary angiograms must be clinically valid (ie, there must be no change in the clinical status or acute coronary syndrome since the qualifying angiogram was performed). Complex PCI is defined as an intervention to the left main stem, requiring calcium modification, or to a chronic total occlusion with a planned retrograde approach or a combination of these. Specific criteria for left main stem intervention and calcium modification are detailed in Figure 3. These criteria were chosen to create a more enriched population than those recruited to BCIS-1 and PROTECT-II, with participants expected to develop substantially more ischemia. CHIP-BCIS3 is expected to have higher event rates than prior trials, improving statistical power and creating greater potential to benefit from elective LV unloading.

Participants can be undergoing PCI for any clinical indication, including stable angina, non–ST-segment–elevation MI or convalescent ST-segment–elevation MI. Exclusion criteria are the presence of cardiogenic shock or acute ST-segment–elevation MI at randomization, contraindication to unloading device insertion (eg, mechanical aortic valve replacement, LV thrombus), inability to give informed consent, or prior enrollment in CHIP-BCIS3 or enrollment in another interventional study that may affect trial outcomes. Myocardial viability testing is at the discretion of the clinical team and not required for inclusion. Monthly screening logs are completed by each center to capture the number of potentially eligible patients who are not randomized and the reason for exclusion, as well as all patients receiving pLVAD-supported PCI regardless of indication. These data will be reported in the primary manuscript CONSORT diagram (Consolidated Standards of Reporting Trials).

Before randomization, it is strongly recommended that all patients undergo assessment of the peripheral vasculature to determine suitability for large-bore vascular access. Assessment may be with computed tomography, ultrasound, or invasive angiography depending on the clinical presentation and physician preference, with a preference for computed tomography. Where significant peripheral vascular disease or access issues are identified, cases must be discussed before randomization in a specialist large-bore access forum (as applicable to each center), which may involve ≥1 of a vascular surgeon, interventional radiologist, or interventional cardiologist with high-volume large-bore access expertise, to formulate a safe access and closure plan.

### Randomization and Treatment

**Figure 4.**
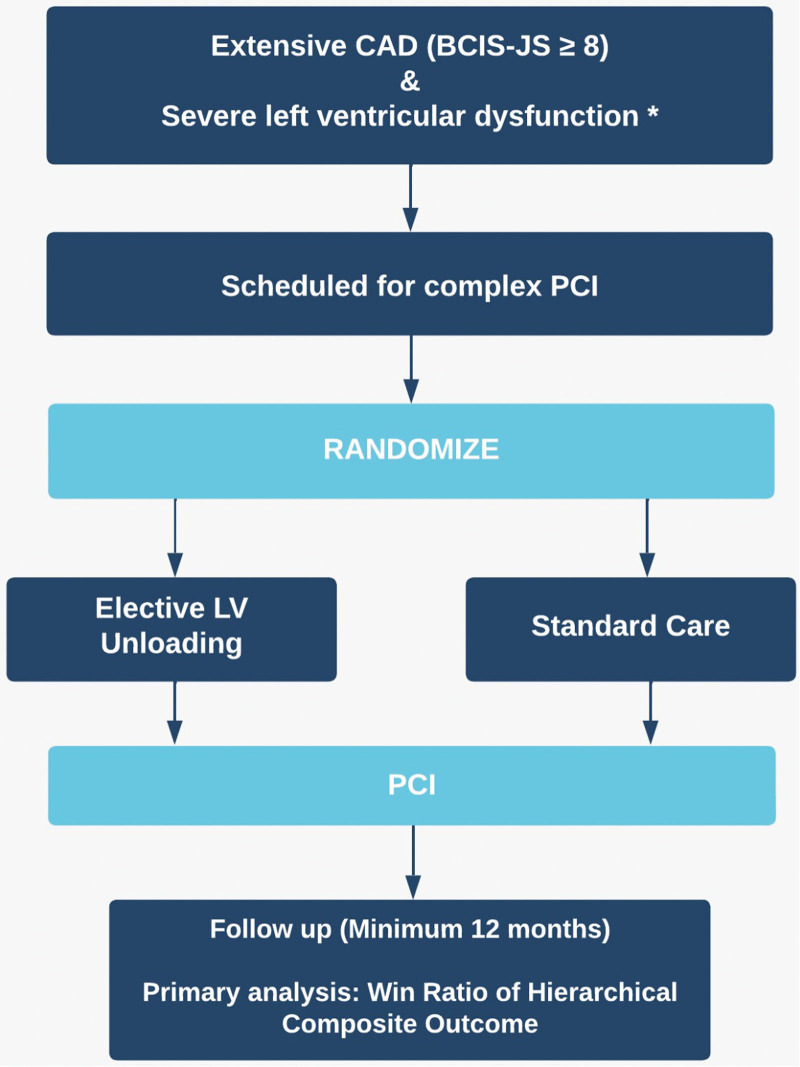
**CHIP-BCIS3 (Controlled Trial of High-Risk Coronary Intervention With Percutaneous Left Ventricular Unloading) flowchart.** BCIS-JS indicates British Cardiovascular Intervention Society Jeopardy Score; CAD, coronary artery disease; LV, left ventricle; and PCI, percutaneous coronary intervention. *Severe LV dysfunction is defined as an LV ejection fraction ≤35% or ≤45% in the presence of severe mitral regurgitation.

Eligible participants who are scheduled to undergo high-risk PCI are randomly assigned in a 1-to-1 ratio to receive either elective LV unloading (LV unloading group) or PCI without planned LV unloading (standard care group; Figure 4). Randomization is via an online randomization system and stratified by center. Participants randomized to the LV unloading group undergo implantation of a pLVAD before the start of PCI and receive maximal support for the duration of the procedure. The choice of pLVAD device is at the discretion of the physician: any CE–marked device intended for the purpose of LV unloading may be used, though the Impella CP is the only device that meets these criteria at present and has been used in all trial cases to date. Femoral arterial access is preferred, but alternative routes (such as axillary or transcaval access) may be utilized where local expertise permits. Use of either ultrasound or fluoroscopy to guide femoral arterial puncture is mandated.

Elective MCS is not permitted in the standard care group. Bailout MCS (with an IABP or venoarterial extracorporeal membrane oxygenation) is permitted only if the patient develops pulmonary edema, cardiogenic shock, profound hypotension, incomplete resolution of mechanical complications of PCI, or cardiac arrest. Where patients in the elective LV unloading group undergo staged PCI, the use of MCS in subsequent procedures is at the discretion of the treating physician. In the standard care group, staged procedures are performed without MCS, unless the previous stage had been complicated by hemodynamic compromise, in which case elective IABP may be used, at the operator discretion.

PCI is performed according to local protocols, although it is recommended that PCI be attempted on all significant coronary lesions in major proximal coronary vessels (or side branches >2.5 mm in diameter) subtending viable myocardium. Physicians are required to identify target lesions before the first PCI procedure and are encouraged to achieve as complete revascularization as they believe to be safe in each procedure. Staging is permitted, in either arm, where operators feel this is the safest way to ultimately achieve complete revascularization, but the intention to stage should be documented before undertaking the first procedure.

### Outcomes

The primary outcome is a hierarchical composite of all-cause death, stroke, spontaneous MI, cardiovascular hospitalization, and periprocedural MI. Outcome definitions are summarized in Table S4. The cardiovascular hospitalization end point is defined as an admission for ≥24 hours with a primary diagnosis of heart failure requiring escalation of therapy, sustained ventricular arrhythmia lasting for ≥30s or causing hemodynamic compromise, or protocol-defined major bleeding or vascular complications. The end point includes prolongation of the index hospitalization subject to the same definitions as rehospitalization. All primary outcome events will be adjudicated by an independent clinical events committee blinded to treatment assignment. Key secondary outcomes include individual components of the primary outcome, completeness of revascularization, major bleeding and vascular complications, health-related quality of life (measured with the EuroQoL-5D-5L [EuroQoL 5 dimension 5 level] and Kansas City Cardiomyopathy Questionnaire instruments), and cost-effectiveness.^46,47^

**Figure 5.**
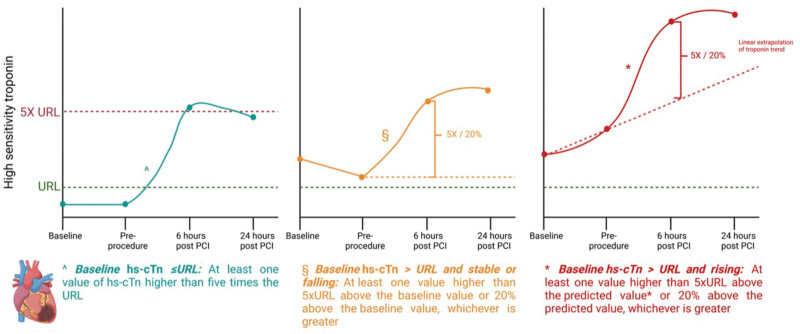
**Definitions of periprocedural myocardial infarction in CHIP-BCIS3 (Controlled Trial of High-Risk Coronary Intervention With Percutaneous Left Ventricular Unloading).** Specific definitions for participants where baseline high-sensitivity cardiac troponin (hs-cTn) is lower than the upper reference limit (URL; **left**), above the URL and stable or falling (**middle**), or above the URL and rising (**right**).

The periprocedural MI definition required modification for a high-risk PCI population to account for participants in whom biomarker levels were elevated and rising at baseline, a situation not accounted for in the Fourth Universal Definition of Myocardial Infarction. Periprocedural MI is defined as the detection of a rise in cardiac troponin I or T, with the threshold of significance defined by the preprocedure value (Table S4; Figure 5). The clinical features required for a diagnosis of periprocedural MI in the Fourth Universal Definition of Myocardial Infarction (new ischemic ECG changes, pathological Q waves, wall motion abnormality, or angiographic evidence of flow-limiting complications) are not included, given evidence that they do not add prognostic value in patients undergoing PCI and blinded review of the angiographic data is rendered impossible by the presence of the pLVAD device. Sensitivity analyses will be performed using the Fourth Universal Definition of Myocardial Infarction, Society for Cardiovascular Angiography and Interventions and Academic Research Consortium-2 definitions, as well as with periprocedural MI excluded.

### Statistical Analysis, Power, and Sample Size

**Figure 6.**
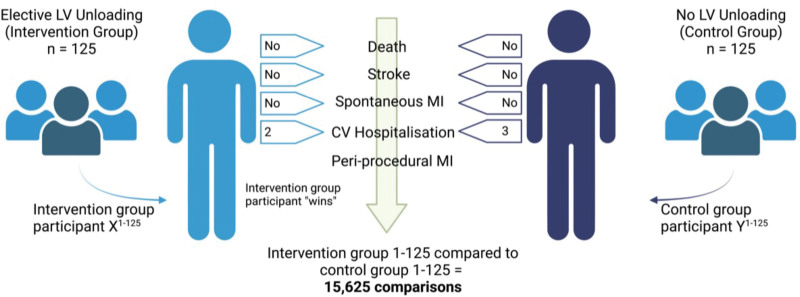
**The win ratio.** Theoretical pair of participants in CHIP-BCIS3 (Controlled Trial of High-Risk Coronary Intervention With Percutaneous Left Ventricular Unloading). In this example, neither participant died or experienced a stroke or spontaneous myocardial infarction (MI). Both patients had a cardiovascular (CV) hospitalization, and as participant X had fewer of these events, they are declared the winner. Periprocedural MI is not considered, as at least 1 participant in the pair experienced a more significant event. LV indicates left ventricle.

A formal Statistical Analysis Plan will be written and signed before database lock, unblinding, and analysis by treatment assignment. All analyses will be on an intention-to-treat basis. Analysis of the primary outcome will be via the unmatched win ratio method, where each participant in one randomized group is compared with every participant in the other randomized group with comparison limited to the shortest duration of follow-up experienced by either individual (Figure 6). The primary analysis will report the overall win ratio and contribution of each component to the outcome.

Where a tie is reached at any tier of the hierarchy, specific criteria are provided to break the tie before moving to the next tier. For all-cause death, stroke, and spontaneous MI, the time to event will be determined and the patient with the longest event-free survival will be the winner. For cardiovascular hospitalization, the patient with fewer hospitalizations during the common period of follow-up will be the winner. For periprocedural MI, the magnitude of infarction will be determined by the absolute change from baseline to the 6-hour high-sensitivity cardiac troponin (hs-cTn) level, expressed as multiples of the upper reference limit of the assay; the patient with the smallest rise in hs-cTn is the winner, unless the difference in increase in hs-cTn is ≤5× the upper reference limit, in which case a tie will be declared.

The win ratio is not invulnerable to all issues with composite end points, in particular, the risk of lower significance events becoming predominant in the analysis if few hard outcomes occur. This issue is unlikely to occur in CHIP-BCIS3, with the recruitment of a population enriched for ischemia and followed up over a longer term, with an expected mortality in excess of 10% per year in the control arm based on observations in BCIS-1 and REVIVED-BCIS2 (Revascularisation for Ischaemic Ventricular Dysfunction).^19,48^ Reporting of the primary outcome will include the proportion of wins at each tier, as well as the proportion of comparisons resulting in a tie. Secondary analyses will be performed for individual components of the primary outcome and for the whole primary outcome using traditional combi time-to-first-event methods, for which the current sample size has an estimated power in excess of 85%. All primary outcome events will be adjudicated by a clinical events committee, to ensure events meet rigorous protocol definitions.

The trial is not designed to conclusively determine the effects of elective pLVAD use on mortality or spontaneous MI alone, though the investigators are keen to conduct individual participant data meta-analyses with other completed and ongoing trials after completion of the primary analysis.

#### Sample Size

The following calculations are based on an assumed accrual period of 3 years and a minimum follow-up of 12 months. As methods for estimation of sample size when using a win ratio approach are still under development, we used 2 methods: first, a conventional approach for a time-to-event composite primary end point and second, based on simulations of the win ratio of different scenarios.

In the PROTECT-II trial, the composite end point comparable to that proposed in CHIP-BCIS3 was 40% at 30 days and 50% at 90 days.^29^ Assuming a more conservative event rate of 50% at 12 months in the control group, a trial of 250 participants (125 in each group) would have at least 80% power to detect a hazard ratio of 0.62 requiring ≈150 first events using all follow-up time (which, at these event rates, represents a risk ratio of 0.70 at 12 months) allowing for 5% losses to follow-up or attrition. This effect size is estimated to be the minimum treatment effect that would need to be demonstrated to justify widespread and routine adoption of a strategy with significant fiscal and clinical cost. Comparable treatment effects have been demonstrated in recent pivotal device trials in interventional cardiology.^49,50^

Based on simulations (examples of which are shown in Table S5), we estimate that ≈65% of patients in the control arm would experience a hierarchical outcome event. Hence, a sample size of 250 would also provide at least 80% power in a win ratio analysis. Though the trial will include fewer participants than prior and ongoing trials of MCS in high-risk PCI, statistical power is determined by the number of events rather than participants; the inclusion of a high-risk population with associated high rates of primary outcome events and novel statistical methods will, therefore, ensure adequate power for definitive results. Blinded event rates will be monitored periodically by the trial steering committee to establish whether the underlying event rate assumptions are accurate and, if lower than predicted, to consider recommending an increase in the sample size.

The protocol does not allow for crossover from standard of care to pLVAD (in this group, bailout if required, should be by IABP or venoarterial extracorporeal membrane oxygenation). Similarly, given the strong recommendation for preprocedure vascular imaging, we anticipate that few patients in the pLVAD arm will have unsuccessful device insertion. Hence, crossovers are expected to be few but will be evaluated throughout the trial.

### Data Collection and Monitoring

Data will be collected using a dedicated online electronic case report form (Sealed Envelope, London, United Kingdom). Baseline data collection includes patient demographics, medical history, British Cardiovascular Intervention Society Jeopardy Score, LV ejection fraction, and details of the coronary anatomy with the intended plan for the PCI procedure. N-terminal pro-B-type natriuretic peptide, hs-cTn, and creatinine levels are obtained before each procedure, with hs-cTn repeated 6 hours after completion of the PCI procedure and again after 24 hours if the patient remains in hospital.

All primary and secondary outcomes will be assessed by research teams 90 days from randomization, at 12 months, and then annually. Corroborating data will also be collected via centrally held NHS electronic health records. All primary outcome and key secondary outcome events (bleeding and vascular complications) will be adjudicated by the Clinical Events Committee, blinded to treatment assignment. At least 2 members of the Clinical Events Committee will review clinical data and other source documentation to determine whether end points have occurred according to the protocol definitions. The Clinical Events Committee charter provides detailed protocols for end point adjudication. Serious and nonserious adverse events will be reported to the sponsor within 7 and 14 days, respectively, and reviewed to establish expectedness and causality. All data will be anonymized and uploaded to an electronic case report form, except for NHS number, which will be encrypted and stored in a separate encrypted database to permit ongoing follow-up via electronic health records. Follow-up will continue until 1 year after the final patient has been randomized, at which point clinical follow-up will conclude and the database will be locked before unblinding of the trial team. Electronic health records will be used for long-term follow-up. A data sharing plan will be published with the trial results.

### Health Economic Analysis

The health economic analysis will be conducted by the Global Health Economics center at the London School of Hygiene & Tropical Medicine. The primary outcome for the cost-effectiveness analysis will be incremental total NHS costs, quality-adjusted life-years, and net monetary benefit at 12 months following randomization; the goal is to show that elective LV unloading is cost-effective compared with the current standard of care.

Resource use data collected through trial electronic case report forms and follow-up questionnaires will be combined with unit costs for the index hospital admission, PCI procedures, MCS devices, and subsequent hospitalizations to report total costs. Health-related quality of life, assessed using the EuroQol-5D-5L questionnaire at 90 days and at 12 months, will be combined with survival data to report quality-adjusted life-years. Baseline health-related quality of life and other patient- and site-level variables will be adjusted for in estimating the adjusted effect of randomization on incremental costs and quality-adjusted life-years. Secondary outcomes for the cost-effectiveness analysis will include resource use, costs, and quality-adjusted life-years at 90 days.

### Involvement of Patients and the Public

Patients and the public are involved throughout. The trial was designed in collaboration with the Guy’s and St Thomas National Institute for Health and Care Research Biomedical Research Centre Patient and Public Cardiac Advisory Group. The trial management group membership includes an experienced patient and public representative, who has contributed substantially to all trial matters. They have had specific roles in designing accessible information in multiple formats to encourage the inclusion of participants from traditionally underrepresented populations. The trial steering committee membership includes 2 independent patient and public representatives, with full voting rights.

### Trial Progress

The trial protocol was approved by the UK Health Research Authority in May 2022 and registered before recruitment of the first participant on August 6, 2021 (https://www.clinicaltrials.gov; unique identifier: NCT05003817 and https://www.isrctn.com; unique identifier: ISRCTN17730734). At the time of publication, 169 participants have been recruited from 21 sites. Recruitment is expected to complete in late 2024. There have been 2 protocol amendments, implemented in January 2023 and August 2023, which clarified the definition of cardiovascular hospitalization and vascular complication, standardized the timing of post-PCI hs-cTn testing, and introduced a minimum difference in hs-cTn rise between patients to determine a win based on periprocedural MI, as well as allowing ongoing clinical follow-up for all participants until the final participant has reached 1 year from randomization. A coronary physiology substudy was also introduced to investigate the effects of randomized assignment to pLVAD support on coronary flow and microvascular resistance at baseline and from pre- to post-PCI, with an intended sample size of 50 participants.

## CONCLUSIONS

The CHIP-BCIS3 trial aims to provide definitive evidence on the role of percutaneous LV unloading devices in patients undergoing high-risk PCI. Specific features of the trial are designed to overcome the limitations of previous randomized trials of MCS in this population, with inclusion criteria enriched for complexity of coronary disease, a hierarchical primary outcome using the win ratio and a longer follow-up period. The trial has been established with 169 participants having been recruited. We anticipate results dissemination in late 2025 or early 2026.

## ARTICLE INFORMATION

### Sources of Funding

This study was funded by the National Institute for Health Research (United Kingdom) Health Technology Assessment Program (NIHR130593).

### Disclosures

None.

### Supplemental Material

Tables S1–S5

## Supplementary Material


