# Supplementary material for: Percutaneous Left Ventricular Unloading During High-Risk Coronary Intervention: Rationale and Design of the CHIP-BCIS3 Randomized Controlled Trial
Source: Circ Cardiovasc Interv. 2024 Feb 27;17(3):e013367. doi: 10.1161/CIRCINTERVENTIONS.123.013367 (PMC10942170; doi:10.1161/CIRCINTERVENTIONS.123.013367)
Supplement: Supplementary file 1 [file hcv-17-e013367-s001.pdf]

Supplemental Material

Table S1 - Observational and randomized comparisons of left ventricular unloading vs. no left ventricular unloading in high-risk PCI

Study	Design	Inclusion Criteria	Number of participants	pLVAD	Control group	Outcomes	Result
Al Khadra et al[54]	Retrospective national registry	ICD9 codes for PCI and Impella or IABP used to identify patients. ACS patients excluded	4,578 Impella, 17,270 IABP	Impella 2.5 or Impella CP	IABP	In hospital mortality<br><br>Vascular complications<br><br>Cardiac complications	Impella patients had lower in hospital mortality 6.1% vs 8.8% p<0.01, vascular complications 4.3% vs 7.5% p =0.046 and cardiac complications 5.6% vs 14.5%, p<0.001)
Amin et al[20]	Retrospective national registry	ICD9/10 codes for PCI and Impella or IABP used to identify patients.	4782 Impella, 43,524 IABP	Impella 2.5, Impella CP or Impella 5.0	IABP	Trends in Impella use<br><br>In hospital mortality<br><br>Bleeding requiring transfusion<br><br>Stroke<br><br>AKI	Impella increased the risk of death (OR 1.24, 95% CI 1.13-1.36), bBleeding (OR 1.10, 95% CI 1.00-1.21) and sStroke (OR 1.34, 95% CI 1.18-1.53)
Azzalini et al[53]	Retrospective single center registry	Any non-emergent pLVAD use	500 patients	Impella 2.5 or Impella CP	Propensity matched controls with no MCS	In hospital and 1 year MACE (all cause death, MI, target lesion revascularization)<br><br>Major bleeding<br><br>Blood transfusion	In-hospital MACE higher in Impella group 26.8% vs 13.2%, p<0.001 whilst no difference observed at 1 year 31.2% vs 27.4%, p=0.78<br><br>Major bleeding 6.7% vs 2.8%, p=0.04<br><br>Blood transfusion 11.2% vs 4.8%, p=0.08
Boudoulas at al[5252]	Retrospective single center registry	LVEF ≤35% and LMS disease or 2 vessel disease	13 Impella, 62 IABP	Impella 2.5	IABP	Vascular complication<br><br>Bleeding	Vascular complication 15.3% Impella vs 6.4% IABP p=0.27.<br><br>Bleeding 38.4% Impella vs 32.2% IABP p=0.74

Lansky et al[2626]	Retrospective national registry	ICD 10 codes used to identify patients undergoing PCI with either Impella or IABP	1447 Impella, 709 IABP	Impella 2.5 or Impella CP	IABP	In hospital survival, MI, cardiogenic shock, stroke, bleeding requiring transfusion and AKI.	In hospital survival 95.3% Impella vs 91.0% IABP p=0.0042. When STEMI excluded survival difference no longer significant p=0.09<br><br>MI 2.5% vs 11.9% p=<0.0001<br><br>Bleeding 2.5% vs 2.3% p=0.88
PROTECT I[5050]	Observational, multicenter prospective study	LVEF ≤35% and either LMS or last patent conduit PCI	20	Impella 2.5	Single arm	30-day MACE (composite of death, MI, TVR, urgent CABG or stroke)	20% experienced MACE
PROTECT II[2525]	Randomized, multicenter trial	LVEF ≤35% and either LMS or last patent conduit PCI<br><br>or<br><br>LVEF ≤30% and 3 vessel disease	452	Impella 2.5	IABP	30-day composite of all cause death, peri-procedural MI, TIA, repeat revascularization, need for cardiac/vascular operation, renal failure, intraprocedural hypotension, CPR, VT, Aortic insufficiency, failure of PCI	Composite end point occurred in 35.1% Impella group vs 40.1% IABP, p =0.227
PROTECT III[5151]	Observational, multicenter prospective study	LVEF ≤35% and either LMS or last patent conduit PCI<br><br>or<br><br>LVEF ≤30% and 3 vessel disease	504	Impella 2.5 or Impella CP	PROTECT II Impella group	90-day MACE (composite of all cause death, stroke/TIA, MI and repeat revascularization)	15.1% of population experienced MACE compared with 21.9% of PROTECT-II control group (p= 0.048)

ACS – acute coronary syndrome, AKI – acute kidney injury, CABG – coronary artery bypass grafting, CI – confidence interval, CPR - cardiopulmonary resuscitation, IABP – intra-aortic balloon pump, LVEF – left ventricular ejection fraction, LMS – left main stem, MACE – major adverse cardiovascular events, MCS – mechanical circulatory support, MI – myocardial infarction, OR – odds ratio, PCI – percutaneous coronary intervention, STEMI – ST-elevation myocardial infarction, TVF – target vessel failure, VT – ventricular tachycardia

Table S2 – Trial Organization

**Committee**	**Members**
Trial Steering Committee	Prof Nick Curzen (Independent Chairperson), University of Southampton<br>Prof Divaka Perera (Chief Investigator), King's College London<br>Prof Tim Clayton (Chief Statistician), London School of Hygiene & Tropical Medicine<br>Dr Rasha Al-Lamee, Imperial College London<br>Dr Adam De Belder, Royal College of Physicians (London)<br>Ms Jacqueline Grudzinskas, Patient and Public representative<br>Prof Jose Henriques, University of Amsterdam<br>Mr Hamid Khan, Patient and Public representative<br>Prof Ly-Mee Yu (Independent Statistician), University of Oxford
Data Monitoring Committee	Prof Rod Stables (Independent Chairperson), Liverpool University<br>Prof Louise Brown (Independent Statistician), University College London<br>Dr Miles Behan, Edinburgh Royal Infirmary<br>Mr Matthew Dodd (Trial Statistician), London School of Hygiene & Tropical Medicine
Trial Management Group	Dr Matthew Ryan, King's College London<br>Mrs Lynn Laidlaw, Patient and Public representative, London School of Hygiene & Tropical Medicine<br>Mr Richard Evans, London School of Hygiene & Tropical Medicine<br>Mr Matthew Kwok, London School of Hygiene & Tropical Medicine<br>Ms Megan Knight, London School of Hygiene & Tropical Medicine<br>Mr Alexander Perkins, London School of Hygiene & Tropical Medicine<br>Dr Saad Ezad, King's College London<br>Prof Tim Clayton<br>Prof Divaka Perera
Clinical Events Committee	Dr Stephen Hoole, Papworth Hospital (Independent Chairperson)<br>Dr Natalia Briceno, Wexham Park Hospital

	Dr Peter Henriksen, Edinburgh Royal Infirmary<br>Dr Rong Bing, Edinburgh Royal Infirmary<br>Dr Abdul Mozid, Leeds General Infirmary<br>Dr Heerajnarain Bulluck, Leeds General Infirmary<br>Dr Paul Bambrough, Royal Papworth Hospital<br>Dr Nicholas Jenkins, Sunderland Royal Hospital

Table S3 – Sites and Investigators

Site	Investigators
Barts Hospital, London	Dr Roshan Weerackody<br>Dr Krishnaraj Rathod<br>Mr Mervyn Andiapen<br>Ms Nasim Forooghi
Bristol Heart Institute, Bristol	Dr Julian Strange<br>Dr Tom Johnson<br>Ms Laura Gallego
Essex Cardiothoracic Centre, Basildon	Dr Tom Keeble<br>Dr John Davies<br>Dr Sarosh Khan<br>Dr Klio Konstantinou<br>Dr Christopher Cook<br>Dr Ozan Demir
Freeman Hospital, Newcastle	Dr Alan Bagnall<br>Dr Adam McDiarmid<br>Ms Kathryn Procter
Glenfield Hospital, Leicester	Dr Andrew Ladwiniec<br>Dr Elved Roberts<br>Ms Jude Fisher
Harefield Hospital, London	Dr Vasileos Panoulas<br>Ms Laura Barker
John Radcliffe Hospital, Oxford	Prof Adrian Banning<br>Dr Jeremy Langrish<br>Dr Giovanni De Maria<br>Ms Vrinda Haridas<br>Ms Bernadette Moreby
King’s College Hospital, London	Dr Ian Webb<br>Dr Matthew Ryan<br>Dr Nilesh Pareek<br>Dr Luke Dancy<br>Dr Rafal Dworakowski<br>Dr Harriet Hurrell<br>Ms Abi Knighton<br>Mr Jon Breeze
Manchester Royal Infirmary, Manchester	Dr Farzin Fath-Ordoubadi<br>Dr Yahya Al-Najjar<br>Ms Stacey Mellor
Morriston Hospital, Swansea	Prof Alex Chase<br>Dr Ahmed Hailan<br>Dr Montasir Ali
Musgrove Park Hospital, Taunton	Dr Mohammad Sahebjalal<br>Dr Thomas Rees<br>Ms Debbie Cutler<br>Ms Michele Swanston
New Cross Hospital Wolverhampton	Dr Ben Wrigley<br>Prof James Cotton<br>Ms Jallen Cruz

Queen Elizabeth Hospital, Birmingham	Dr Sohail Khan<br>Prof Peter Ludman<br>Ms Annette Nilsson
Royal Bournemouth Hospital, Bournemouth	Dr Peter O'Kane<br>Dr John Rawlins<br>Dr Jonathan Hinton<br>Ms Sarah Orr<br>Ms Stephanie Horler
Royal Brompton Hospital, London	Dr Ranil De Silva<br>Dr Jonathan Hill<br>Dr Kevin Cheng<br>Dr Lap Tin Lam<br>Ms Carmen Chan
Royal Cornwall Hospital, Truro	Dr Tamas Ungvari<br>Ms Keren Northcott<br>Ms Katie Morgan<br>Ms Sue Webber
Royal Sussex County Hospital, Brighton	Dr Michael Michail<br>Dr Christopher Broyd<br>Ms Nicola Skipper
Royal Victoria Hospital, Belfast	Dr Colum Owens<br>Dr Andrew McNiece<br>Ms Patricia Glover<br>Ms Sharon Nelson
St George's University Hospital, London	Dr Claudia Cosgrove<br>Prof James Spratt<br>Dr Rupert Williams<br>Ms Vennessa Sookhoo
St Thomas' Hospital, London	Prof Divaka Perera<br>Dr Matthew Ryan<br>Dr Kalpa De Silva<br>Dr Antonis Pavlidis<br>Dr Haseeb Rahman<br>Mr Ash Patel<br>Dr Saad Ezad<br>Dr Holly Morgan<br>Dr Matthew Li Kam Wa<br>Ms Amy Raynsford<br>Ms Sophie Arnold<br>Ms Stephanie Hunt

Table S4 – Outcome definitions

**Primary Outcome**	
Combined hierarchical endpoint incorporating death, disabling stroke, spontaneous myocardial infarction, cardiovascular hospitalization and periprocedural myocardial infarction, analyzed with the win ratio method.	
**Components of the primary outcome**	
Death	All-cause mortality
Stroke	Stroke is defined as an acute episode of focal or global neurological dysfunction caused by brain, spinal cord, or retinal vascular injury as a result of hemorrhage or infarction, resulting in persistent moderate disability (modified Rankin Scale ≥3) at the time of discharge from the acute hospital admission.
Spontaneous myocardial infarction (> 48 hours after PCI or CABG)	Detection of a rise and/or fall of cardiac Troponin I or T, with at least one value higher than the 99th percentile upper reference limit (URL) AND symptoms consistent with ischemia OR dynamic electrocardiogram (ECG) changes (including ≥1mm ST elevation or ST depression, new left bundle branch block (LBBB) or >3mm T-wave inversion) OR imaging evidence of new loss of viable myocardium or new regional wall motion abnormality in a pattern consistent with an ischemic etiology.
Cardiovascular hospitalization	• Hospital admission (lasting ≥24 hours) with a primary diagnosis of heart failure or sustained ventricular arrhythmia. Prolonged hospitalization for complications of the PCI procedure: acute heart failure, major bleeding and major vascular complication are included within the definition where the length of admission is extended by ≥24 hours from the expected time of discharge following the procedure and the associated endpoint definition has been met and was the primary reason for prolongation of the hospital admission.<br>• Heart failure hospitalization will be defined as Hospital admission (lasting >24 hours) for deteriorating symptoms or signs of heart failure, where there is a documented diagnosis of heart failure and the patient receives initiation or intensification of treatment for heart failure. Initiation or intensification of treatment includes at least one of the following: increase in oral diuretic dose or addition of another oral diuretic; intravenous diuretic therapy;

	intravenous vasoactive therapy (vasodilator, inotrope or vasopressor); mechanical circulatory support (MCS) (including intra-aortic balloon pump (IABP), pLVAD, extra-corporeal membrane oxygenation (ECMO)); or cardiac transplantation.<br>• Heart failure during or after the assigned PCI procedure itself is defined as prolongation of the planned admission by at least 24 hours due to acute heart failure requiring initiation or intensification of treatment as defined above (including continued use of pLVAD for >24hours after PCI in patients randomize to the elective LV unloading group, for a clinical suspicion of heart failure). Elective admission for implantation or revision of ICD/cardiac resynchronization therapy (CRT) devices will NOT constitute an endpoint.<br>• Sustained ventricular arrhythmia is defined as Ventricular tachycardia or fibrillation persisting for more than 30 seconds and/or associated with hemodynamic compromise, and/or requiring cardioversion/defibrillation (external or via implantable cardioverter defibrillator). Suspicion of arrhythmia without documentation on a recorded surface ECG or electrograms from an indwelling device will not constitute an endpoint.<br>• Elective admission for planned cardiac procedures (staged PCI, device insertion, cardioversion or catheter ablation) will not constitute an endpoint.
Periprocedural myocardial infarction (< 48 hours after PCI/CABG)	• **Following PCI:** Detection of a rise in cardiac troponin I or T, with the threshold of significance determined by the pre-procedure baseline value.<br>• ***Baseline ≤URL:*** At least one value higher than five times the URL

	• ***Baseline > URL and stable or falling:*** At least one value higher than 5xURL above the baseline value or 20% above the baseline value, whichever is greater.<br>• ***Baseline > URL and rising:*** At least one value higher than 5xURL above the predicted value* or 20% above the predicted value, whichever is greater.<br>• **the predicted value will be calculated via linear extrapolation of the trend from at least two troponin values taken within 48 hours before the procedure.*<br>• **Following CABG:** As for PCI, but with a threshold of 10xURL.<br><br>*In addition to classifying patients dichotomously as having suffered a periprocedural MI or not, baseline and peak troponin I or T values measured within 24 hours of a procedure will be recorded. This will provide a continuous measure for adjudication of ties in patients reaching the periprocedural myocardial infarction endpoint within the win ratio.*<br><br>*Absolute values of troponin, ECGs and supporting information will be collected for all patients who experience a periprocedural MI, so that sensitivity analyses based on alternative definitions can be explored.*
**Major secondary outcomes**	

Major bleeding	Major bleeding will be defined using the Bleeding Academic Research Consortium (BARC) categories below:<br>**Type 3: Major Bleeding**<br>***Type 3a***<br>• Overt bleeding plus hemoglobin drop of ≥30 to <50g/L (provided hemoglobin drop is related to bleed)<br>• Any transfusion with overt bleeding<br>***Type 3b***<br>• Overt bleeding plus hemoglobin drop ≥50g/L (provided hemoglobin drop is related to bleed)<br>• Cardiac tamponade<br>• Bleeding requiring surgical intervention for control (excluding dental/nasal/skin/hemorrhoid)<br>• Bleeding requiring intravenous vasoactive drugs<br>***Type 3c***<br>• Intracranial hemorrhage (does not include microbleeds or hemorrhagic transformation; does include intraspinal)<br>• Subcategories; confirmed by autopsy, imaging or lumbar puncture<br>• Intra-ocular bleed compromising vision<br>**Type 4: CABG-Related Bleeding**<br>• Perioperative intracranial bleeding within 48 hours<br>• Reoperation following closure of sternotomy for the purpose of controlling bleeding<br>• Transfusion of ≥5 units of whole blood or packed red blood cells within a 48-hour period<br>• Chest tube output ≥2L within a 24-hour period<br>• If a CABG-related bleed is not adjudicated as at least a Type 3 severity event, it will be classified as 'Not a bleeding event'<br>**Type 5: Fatal Bleeding**<br>***Type 5a***<br>• Probable fatal bleeding: no autopsy or imaging confirmation, but clinically suspicious<br>***Type 5b***<br>• Definite fatal bleeding: overt bleeding or autopsy or imaging confirmation

Vascular complication	Vascular complications will be defined according to the valve academic research consortium (VARC) criteria below:<br>**Major complication**<br>• Aortic dissection or aortic rupture<br>• Vascular (arterial or venous) injury# or compartment syndrome resulting in death, VARC type ≥2 bleeding, limb or visceral ischemia, or irreversible neurologic impairment<br>• Distal embolization (non-cerebral) from a vascular source resulting in death, amputation, limb or visceral ischemia, or irreversible end-organ damage<br>• Unplanned endovascular or surgical intervention resulting in death, VARC type ≥2 bleeding, limb or visceral ischemia, or irreversible neurologic impairment<br>• Closure device failure resulting in death, VARC type ≥2 bleeding, limb or visceral ischemia, or irreversible neurologic impairment<br>**Minor complication**<br>• Vascular (arterial or venous) injury[#] *not* resulting in death, VARC type ≥2 bleeding, limb or visceral ischemia, or irreversible neurologic impairment<br>• Distal embolization treated with embolectomy and/or thrombectomy, *not* resulting in death, amputation, limb or visceral ischemia, or irreversible end-organ damage<br>• Any unplanned endovascular or surgical intervention, ultra-sound guided compression, or thrombin injection, *not* resulting in death, VARC type ≥2 bleeding, limb or visceral ischemia, or irreversible neurologic impairment<br>• Closure device failure not resulting in death, VARC type ≥2 bleeding, limb or visceral ischemia, or irreversible neurologic impairment
Major procedural complication	• VT/VF requiring defibrillation.<br>• Cardiorespiratory arrest or acute pulmonary oedema requiring assisted ventilation.<br>• Prolonged hypotension (Mean arterial pressure ≤75 mmHg for >10 min despite fluid resuscitation and/or vasoactive drugs and/or requirement of mechanical circulatory support)

Acute kidney injury	Acute kidney injury defined as prolonging hospital admission or readmission ≥ 24 hours with rise in creatinine to 200% of baseline value or need for new renal replacement therapy within 30 days of procedure.
Completeness of revascularization	Change in anatomic BCIS-JS and anatomic SYNTAX score between the time of randomization and the completion of the final planned PCI procedure.

CABG – coronary artery bypass grafting, PCI – percutaneous coronary intervention, URL – upper reference limit, VT – ventricular tachycardia, VF – ventricular fibrillation

Table S5 – An example of a simulated power calculation by the win ratio analysis

**Simulation Assumptions**
Length of follow-up
Minimum follow-up time = 1 year, maximum follow-up time = 4 years
Event rates amongst the control group
Death rate of 10% in first year, followed by 4% per year<br>Stroke rate of 2% in first year, followed by 1% per year<br>Spontaneous MI rate of 4% in first year, followed by 2% per year<br>CV hospitalization rate of 20% per year<br>Peri-op MI prevalence of 20%
Treatment effect
Hazard ratio for the effect of LV unloading assumed to be 0.62 for each time-to-event component of the outcome<br>Risk ratio for the effect of LV unloading assumed to be 0.70 for the peri-op MI component (equivalent to a hazard ratio of 0.62).
